# COVID‐19‐related rhino‐orbital‐cerebral mucormycosis: Neurological manifestation and related risk factors in two cases

**DOI:** 10.1002/ccr3.6352

**Published:** 2022-10-20

**Authors:** Mohammad Amin Dabbagh Ohadi, Gelareh Banihashemi, Nader Akbari Dilmaghani, Mohammad Ali Shamsi, Guive Sharifi

**Affiliations:** ^1^ Skull Base Research Center, Loghman Hakim Hospital Shahid Beheshti University of Medical Sciences Tehran Iran; ^2^ Department of Neurology, Sina Hospital Tehran University of Medical Sciences Tehran Iran; ^3^ Students' Scientific Research Center Tehran University of Medical Sciences Tehran Iran; ^4^ Hearing Disorders Research Center, Loghman Hakim Hospital Shahid Beheshti University of Medical Sciences Tehran Iran; ^5^ Department of Otolaryngology, Head and Neck Surgery, Loghman Hakim Educational Hospital, School of Medicine Shahid Beheshti University of Medical Sciences Tehran Iran; ^6^ Department of Neurological Surgery, Loghman Hakim Hospital, Faculty of Medicine Shahid Beheshti University of Medical Science Tehran Iran; ^7^ Professor of Neurosurgery, Skull Base Research Center, Loghman Hakim Hospital Shahid Beheshti University of Medical Sciences Tehran Iran

**Keywords:** COVID‐19, diabetes mellitus, ROCM, seizure

## Abstract

Mucormycosis is an opportunistic infection that has become a serious concern as a result of the immunosuppressive drugs used during COVID‐19. In this report, we describe two cases of rhino‐orbital‐cerebral mucormycosis with neurological presentation and ophthalmologic problems accompanied by a history of COVID‐19 and diabetes.

## 1 INTRODUCTION

Mucormycosis is an opportunistic infection caused by organisms belonging to the order Mucorales affecting between 0.4 and 1.7 cases per 10,000 of the world's population as reported in 2012.^1^, ^2^ Due to COVID‐19, mucormycosis has become far more common and is now considered a major health threat.^1^, ^3^ Rhino‐orbital‐cerebral mucormycosis (ROCM) is the common type of mucormycosis mostly associated with diabetes mellitus (DM, particularly uncontrolled DM) and less commonly other immunosuppressive conditions such as malignancies, organ transplants, HIV, and long‐term broad‐spectrum antibiotics or immunosuppressive treatment like steroids which is the conventional treatment for COVID‐19.^4^

Despite recent case reports and systematic reviews, we still do not have a strong grasp of mucormycosis, including the possibility of brain invasion, abscess formation, and neurological symptoms. Unfortunately, Bhattacharyya et al. reported a high mortality rate of mucormycosis related to COVID‐19 (33%–80%).^1^ in this article, we will discuss two cases of ROCM with brain abscesses, unusual neurological presentations, and possible effective management.

## 2 CASES

### 2.1 Case 1

40 Y/O man came to the emergency room with a seizure, reduced level of consciousness (LOC), and headache. In the last 5 days, he experienced fever and malaise. On examination, there was mild ptosis with restricted movement toward the right in the right eye suggesting right abducens nerve damage accompanied by impaired visual acuity and color vision. There was not any sign of palate necrosis or nasal discharge. Kernig sign was negative; however, specimen from lumbar puncture evidenced meningitis.

**FIGURE 1:**
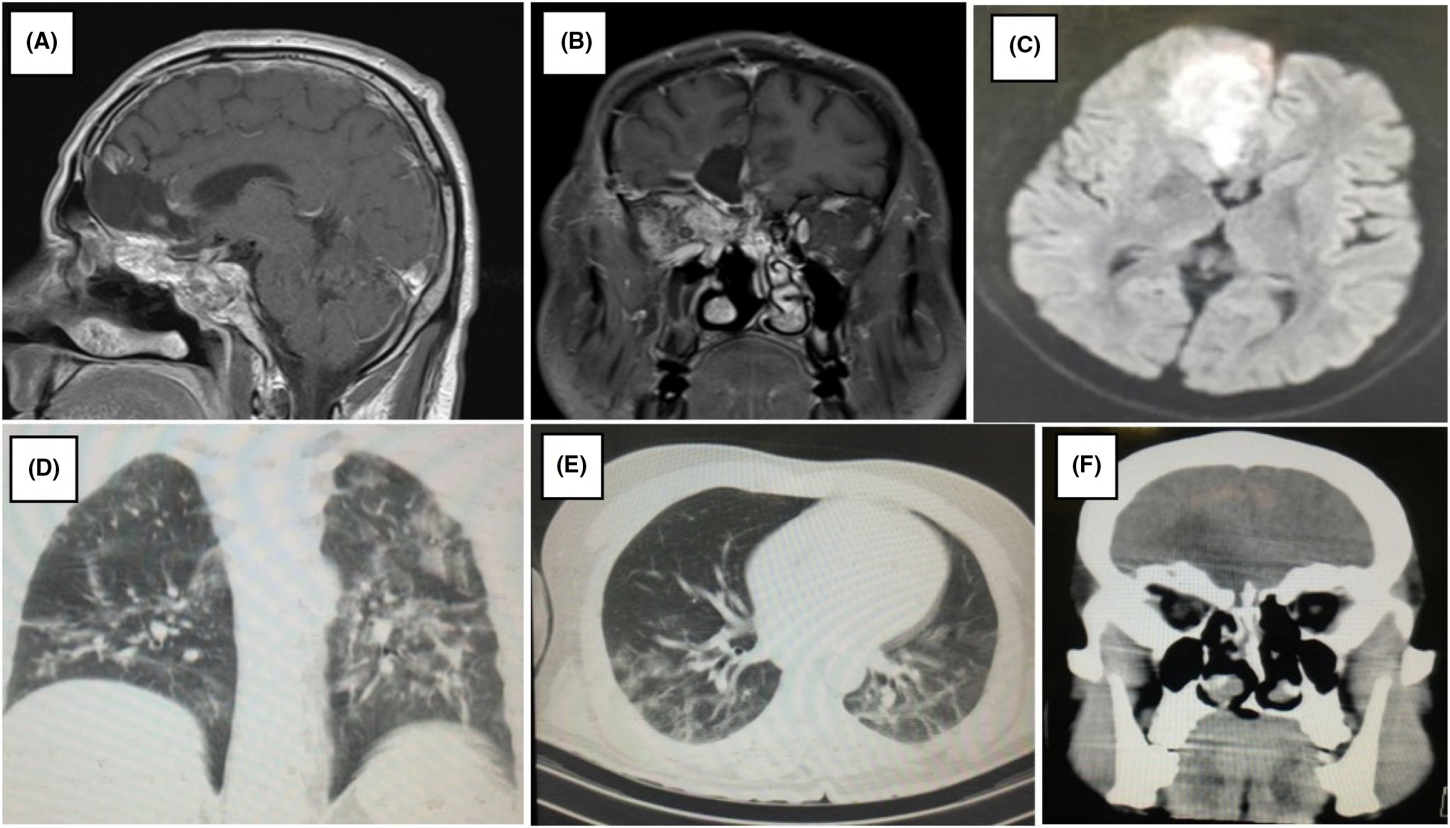
A, B, C: The parenchymal lesion in left frontal base with mild enhancement paranasal sinus involvement in T1 with D, E: Chest CT scan illustrating COVID‐19 involvement F: hypodensity in left frontal base.

Patients declared a history of diabetes with regular insulin usage and COVID‐19 infection in the past 2 months. A Chest CT scan denoting an ongoing COVID‐19 infection was obtained (Figure 1). The patient had experienced uncontrolled blood sugar (Blood Sugar = 548 mg/dl) during the usage of broad‐spectrum antibiotics along with corticosteroids at the time of the COVID‐19 infection. We also recorded a rise in creatinine (highest blood creatinine = 3.3 mg/dl) during treatment.

Imaging evaluation revealed CNS findings as depicted in Figure 1. Suggesting mucormycosis or other bacterial infection mimicking low‐grade glioma; therefore, we started broad‐spectrum antibiotics accompanied by liposomal amphotericin B (5 mg/kg) followed by definitive surgery.

There were no sinonasal symptoms but due to the origin of the lesion that was seen from below, we chose the Endoscopic transnasal corridor. After ethmoidectomy, we encountered frank puss and granulation tissue as white creamy material. The grand and basal lamellae were destroyed. We entered the cranial cavity; a Frontal basal abscess was drained and copious irrigation was implemented. We took advantage of the absence of arachnoid bridge and CSF leakage and let the skull base be opened to the nasal cavity for further abscess drainage.

Despite our expectations for suppurative bacterial infection, pathological findings were as follows:

Section of brain tissue shows well‐formed and occasionally coalescing granulomas composed of epithelioid histiocytic multinucleated giant cells, lymphocytes, and central neutrophilic microabscesses with an area of necrosis containing fragmented broad fungal hyphae with right‐angle branching and no septation, perivascular inflammation, astrogliosis, and edema are noted which is diagnosed as necrotizing granulomatous encephalitis consistent with mucormycosis without any evidence of malignancy.

In the course of treatment, the patient's headache and drowsiness worsened. New imaging revealed aggravation of cerebral edema and persistent frontal mass effect. Hence, unilateral frontal craniotomy and removal of necrotic tissues were performed, and it was attempted to close the base defect with fascia lata and decompress the frontal lobe.

After the surgery, three sessions of retrobulbar amphotericin B (5 mg/kg) were injected by ophthalmologists due to orbital apex syndrome (OAS). Nasal endoscopy was normal before discharge. Finally, the patient was discharged after 2 months without any visual deficit or headache. He was prescribed to use posaconazole (300 mg) and antibiotics. After 3 months of follow‐up, patient has not experienced any significant neurological sing and symptoms including headache, altered LOC, and visual defects along with any recurrence manifestation.

### 2.2 Case 2

Our second case is a 50Y/O man who presented with seizures (2 times), headache, obtundation, left mild proptosis, and orbital pain since 2 weeks ago; 3 weeks after recovery from COVID‐19 infection. At the physical examination, we did not find further remarkable neurological signs.

The patient had a history of diabetes mellitus. COVID‐19 was confirmed by PCR and corticosteroid usage was noted in this treatment regimen. Based on the probability of mucormycosis, we started liposomal IV amphotericin B (5 mg/kg) and broad‐spectrum antibiotics followed by the surgery.

Although MRI defined intracranial mass and edema, because of the involvement of ethmoid sinus and continuation of epidural collection to sinonasal space, we had a preliminary diagnosis of purulent complicated sinusitis with epidural empyema and chose the intranasal endoscopic path to open affected sinuses and brain epidural puss and irrigation. We found black debris and puss characteristics of sinonasal mucormycosis. Ethmoidectomy and entrance to epidural space ensued.

Despite the specimen and puss was not confronting a classic fungal mucormycosis infection, the pathology was as follows: Left periorbital soft tissue debridement is necrotic and inflamed fibro adipose tissue with non‐septate right‐angle branching hyphae which is morphologically consistent with mucormycosis fragments of inflamed and necrotic respiratory mucosa present middle turbinate and bilateral ethmoidal sinus debridement, focally necrotic respiratory mucosa with non‐necrotizing granulomatous inflammation no fungal element identified in this sample.

**FIGURE 2:**
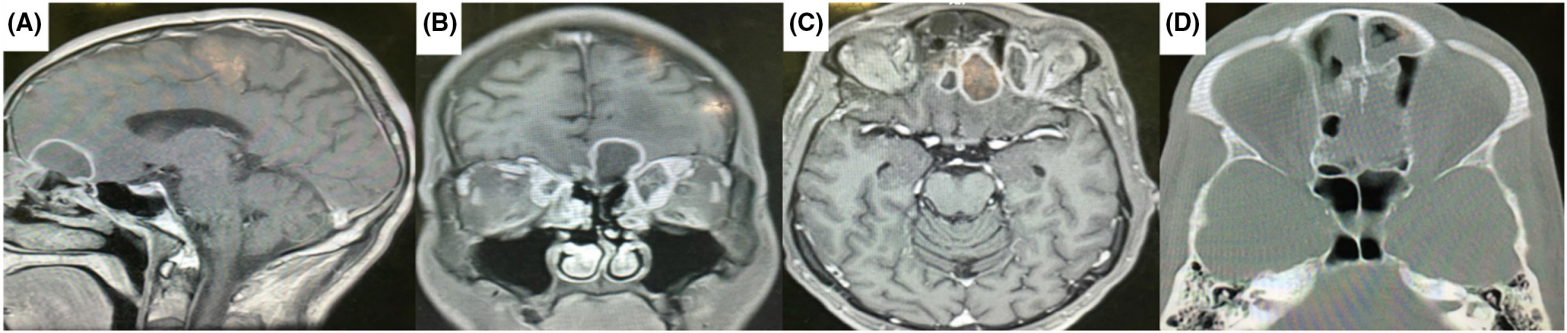
(A–C) Hypointense lesion in T1 with ring enhancement in left anterior skull base and frontal base which spreads to the medial part of left orbit (D) ethmoidal erosion in bone window CT scan.

A week after surgery, sinus endoscopy revealed septum necrosis, so we resected the crust, and the sinuses were washed. Patient's diabetes was controlled by our endocrinologist during the treatment course, and finally after one month, patient was discharged from the hospital without visual defect or headache. During the last 3 months of follow‐up, there is no evidence of significant neurological problems as well as any recurrence of the infection (Figure 2).

## 3 DISCUSSION

Rhino‐cerebral mucormycosis is an aggressive infection and failure to diagnose or treat can lead to high mortality.^1^ Mucormycosis has five different spread sites: rhino‐cerebral, gastrointestinal, cutaneous, respiratory, and disseminated. ROCM is the predominant manifestation of the infection which can progress to the orbital and deeper sites of the brain through paranasal sinuses^1^, ^5^ and could result in uncommon presentations such as seizures, reduced LOC, or ophthalmic involvement. Mucormycosis infects the nervous system in 20% of hematological cases and brain abscess occurs rarely in 2 of 43 patients with mucormycosis.^5^, ^6^ Unfortunately, it has been observed that mucormycosis has been rising since the COVID‐19 pandemic began, and recently it has become an issue of concern for our healthcare system.^1^ The condition could be a consequence of a dysregulated immune system due to a reduction in T cell count, altered CD4/CD8 ratio, and overuse of immunosuppresses like corticosteroids following the COVID‐19 which is exacerbated by uncontrolled blood sugar in people with undetermined diabetes cases.^1^ Sharma et al. in their prospective study believe diabetes is the most common risk factor for the ROCM after COVID‐19 which 57% of the cases presenting with uncontrolled diabetes (HBA1c > 6.5 mg/dl).^7^

Diagnosis of ROCM is based on the clinical finding including headache, nasal or sinus congestion, and blackish discoloration within the nose or palate. Radiological findings such as CT or MRI and pathological investigations are crucial for confirmation of the diagnosis. Involvement of the paranasal sinuses and at the top of them ethmoid sinus is the highly common form. However, it could progress to the other sinuses and infect the orbit, extraocular muscles, and finally, the brain.^8^

Rhinorage and the blackish plate were the most common presentation of mucormycosis in our center during COVID‐19; however, the common presentation was absent in our recent cases. Instead, these two patients suffered from neurological problems such as seizures, low LOC, and OAS, which means we should be concerned more about mucormycosis in immunosuppressed patients, especially in tumor mimicking masses, and always keep it in our differential diagnosis.

Visual defects were reported in mucormycosis cases, this may be caused by infarctions in blood vessels supplying the retina or optic nerve, in addition to the compression of the nerve through the cavernous sinus, as well as infection and necrosis.^8^

Amphotericin B plus posaconazole was the regimen commonly used in previous studies.^1^ Consistent with our cases, other studies found intraorbital and retrobulbar injection of amphotericin B useful.^1^, ^9^ However, surgical debridement is the final and crucial approach for these patients.

## 4 CONCLUSION

We believe the incidence of this opportunistic infection is increasing due to the higher incidence of the mentioned risk factors and the key solution was early detection since mucormycosis has broad manifestation and correct intervention to reduce mortality and morbidity.

## AUTHOR CONTRIBUTIONS

GS, NAD, and MAS were involved in the interpretation and collecting of data and editing of the manuscript. MADO was involved in drafting the first version of the manuscript and editing. GB was involved in collecting data and editing and submitting the manuscript. All authors reviewed the paper and approved the final version of the manuscript.

## FUNDING INFORMATION

There is no funding source for authors to declare.

## CONFLICT OF INTEREST

None declared.

## ETHICAL APPROVAL

The study was approved by our local ethics committee.

## CONSENT

Written informed consent was obtained based on the journal's patient consent policy.

## Data Availability

The data that support the findings of this study are available from the corresponding author upon reasonable request.
